# The Cure of Strabismus by Surgical Operation

**Published:** 1841-10

**Authors:** 


Art. XII.—*The Cure of Strabismus by Surgical Operation.* By W. Mackenzie, m.d., &c.—*London,* 1841. 8vo, pp. 30.

This pamphlet is an Appendix to the author's "Practical Treatise on the Diseases of the Eye," and contains, in a condensed form, a complete account of all that is at present known on the subject of which it treats. We cannot pay it a higher compliment than to say that it is written in the same admirable style as the work to which it is a well-timed and fitting supplement.

